# Supplementary material for: Brominated lipid probes expose structural asymmetries in constricted membranes
Source: Nat Struct Mol Biol. 2023 Jan 9;30(2):167–75. doi: 10.1038/s41594-022-00898-1 (PMC9935397; doi:10.1038/s41594-022-00898-1)
Supplement: Supplementary file 1 — Supplementary Figs. 1–7, Tables 1–8, Discussion and Protocols. [file 41594_2022_898_MOESM1_ESM.pdf]

nature structural & molecular biology

Article https://doi.org/10.1038/s41594-022-00898-1

Brominated lipid probes expose structural asymmetries in constricted membranes

In the format provided by the authors and unedited

Supplementary Information

Brominated Lipid Probes Expose Structural Asymmetries in Constricted Membranes

Frank R. Moss III,[1,2,8†] James Lincoff,[3,4†] Maxwell Tucker,[3,4] Arshad Mohammed,[1,5,8] Michael Grabe,[3,4*] Adam Frost[1,6,7,8*]

[1] Department of Biochemistry & Biophysics, University of California San Francisco; San Francisco, CA 94158, USA. [2] SLAC National Accelerator Laboratory, Menlo Park, CA 94025 [3] Department of Pharmaceutical Chemistry, University of California San Francisco; San Francisco, CA 94158, USA. [4] Cardiovascular Research Institute, University of California San Francisco; San Francisco, CA 94158, USA. [5] University of California Berkeley; Berkeley, CA 94720, USA. [6] Chan Zuckerberg Biohub; San Francisco, CA 94158, USA. [7] Department of Biochemistry, University of Utah, Salt Lake City, UT 84132, USA.

[8] Present address: Altos Labs, Redwood City, CA 94065

* Corresponding authors. Email: afrost@altoslabs.com (A.F.) and michael.grabe@ucsf.edu (M.G.).

† These authors contributed equally to this work.

1. NMR and mass spectra S2
2. Simulation Analysis S10
3. Cryo-EM imaging and data processing S13
4. Analysis of leaflet compositions S16
5. Cryo-EM Data for all Brominated Lipid Samples S17
6. Negative stain TEM S18
7. Effects of data collection and analysis parameters S19
8. Effects of varying cholesterol concentration S20
9. Sub-stoichiometric SDPC-Br S21
10. CHMP1B Phe mutants S22
11. 19-Iodocholesterol S23
12. Pressure-area isotherms of lipids and bromolipids S24
13. CHMP1B-IST1 Composition 1 Tubule Simulation Setup and Parameters S25
14. Experimentally Derived Lipid Composition with WT CHMP1B-IST1 Simulations S27
15. Supplementary simulation data S28
16. References S30

1. NMR and mass spectra

The identity of brominated lipids and the extent of bromination were assessed with ^{13}C and ^{1}H NMR for cholesterol, POPS, and SDPC and ESI-MS for cholesterol, POPS, SDPC, and PIP_2. The spectra are shown below in Fig. S1. No purification of brominated lipids was performed in order to maintain an accurate total quantity of lipid. As a result, some of the brominated lipids contain unbrominated starting material and/or partially brominated lipids. For this application, where relative amounts of lipids in the vesicles is critical, we believe this approach is preferable to further purifying the brominated lipids and attempting to measure milligram quantities with high uncertainty. To quantify the extent of bromination, peak intensities for brominated and unbrominated species in MS and integrated peak areas for brominated and unbrominated species in NMR were measured. The extent of bromination was calculated as the average number of Br atoms per molecule for each lipid.

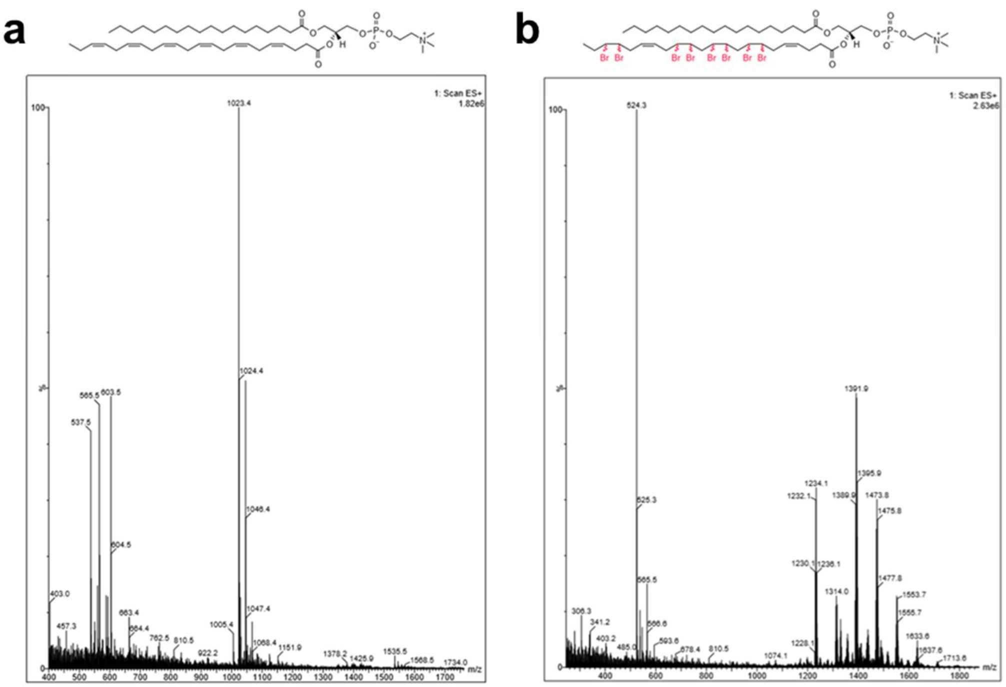

c

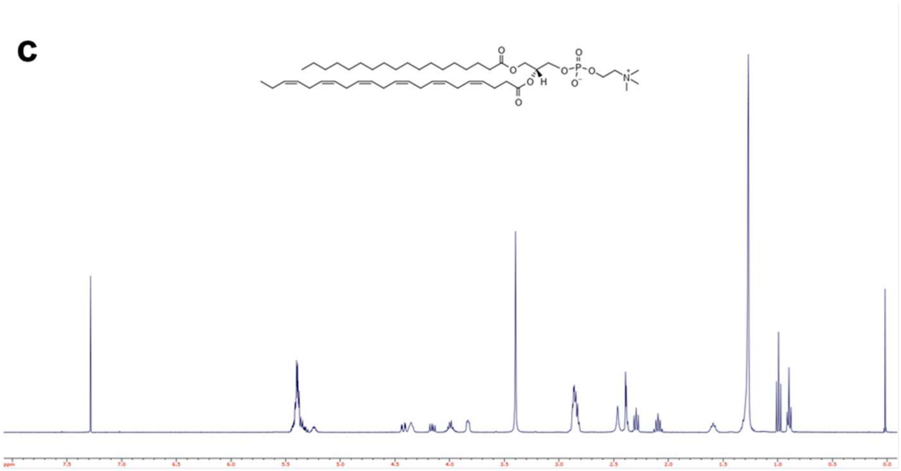

d

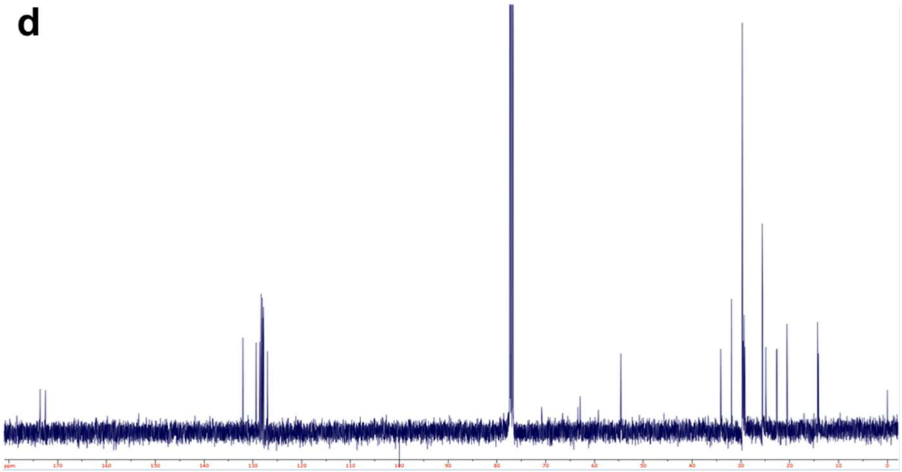

e

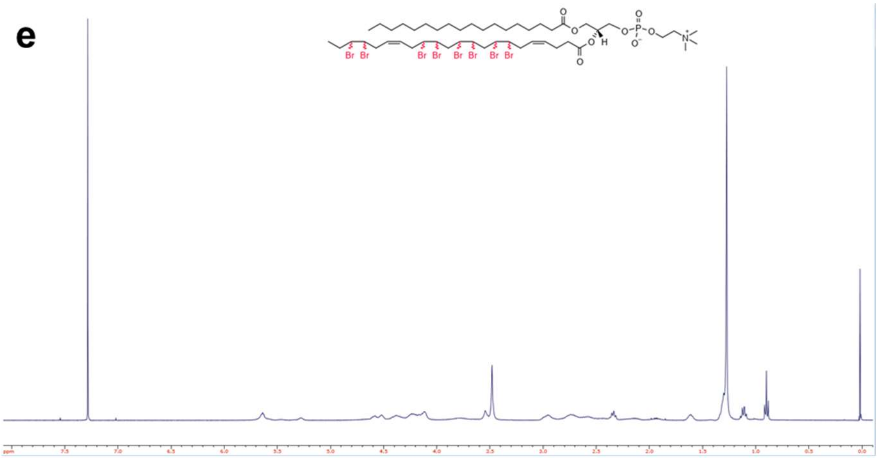

Br Br Br Br Br Br Br Br

f

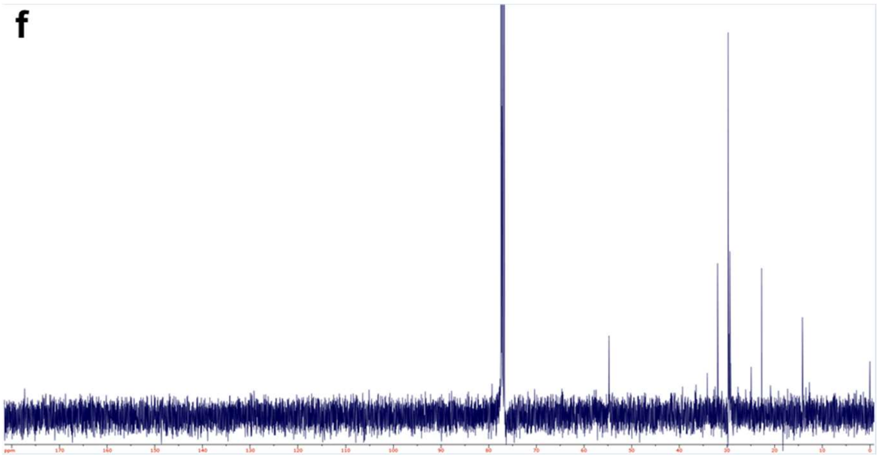

g

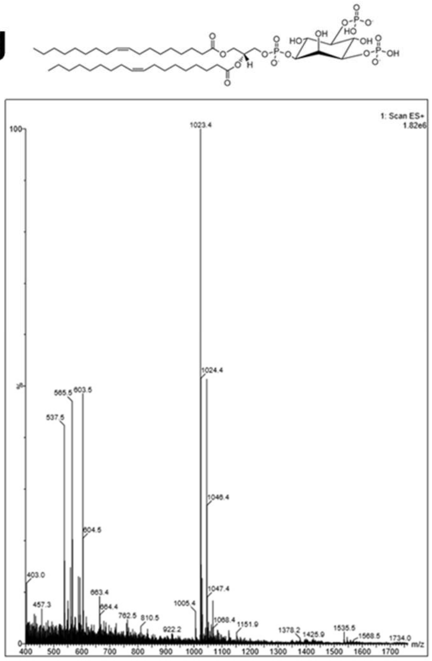

1: Scan ES+
1.82e6
1023.4
1024.4
565.5
603.5
537.5
1046.4
604.5
403.0
663.4
457.3
664.4
762.5
810.5
922.2
1005.4
1047.4
1068.4
1151.9
1378.2
1425.9
1535.5
1568.5
1734.0
m/z

h

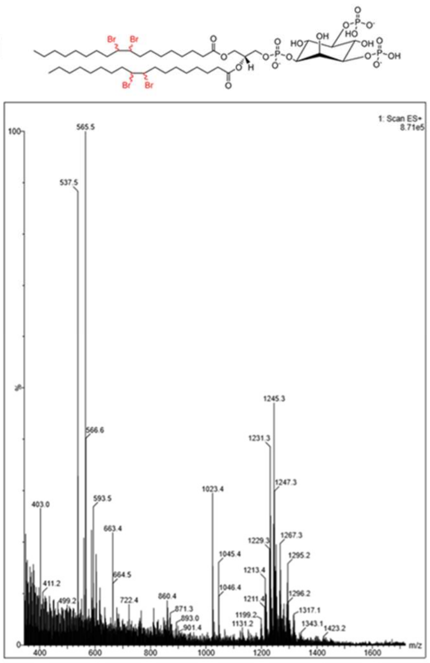

Br Br
Br Br
1: Scan ES+
8.71e5
565.5
537.5
1245.3
566.6
1231.3
1247.3
593.5
403.0
1023.4
663.4
1229.3
1267.3
1295.2
1045.4
1213.4
664.5
411.2
1046.4
1211.4
722.4
860.4
1296.2
499.2
871.3
1317.1
1199.2
893.0
1343.1
901.4
1131.2
1423.2
m/z

i

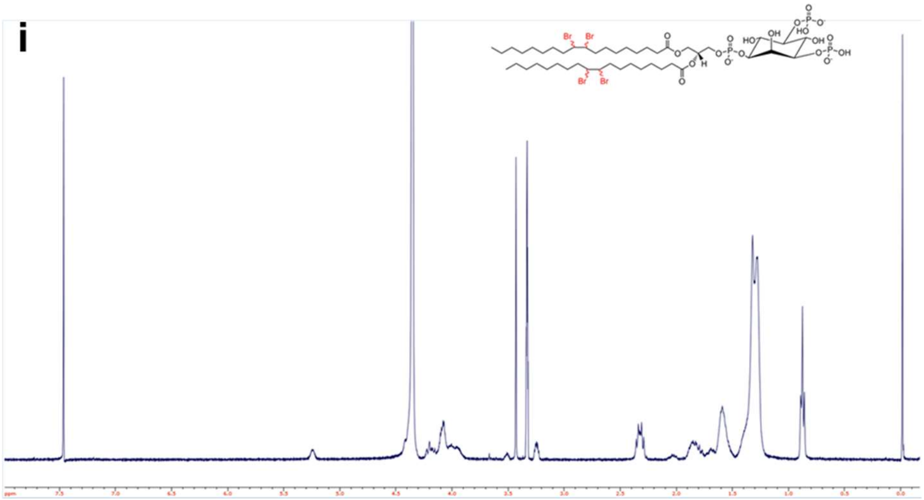
Br
Br
HO
OH
OH
ppm
7.5
7.0
6.5
6.0
5.5
5.0
4.5
4.0
3.5
3.0
2.5
2.0
1.5
1.0
0.5
0.0

j

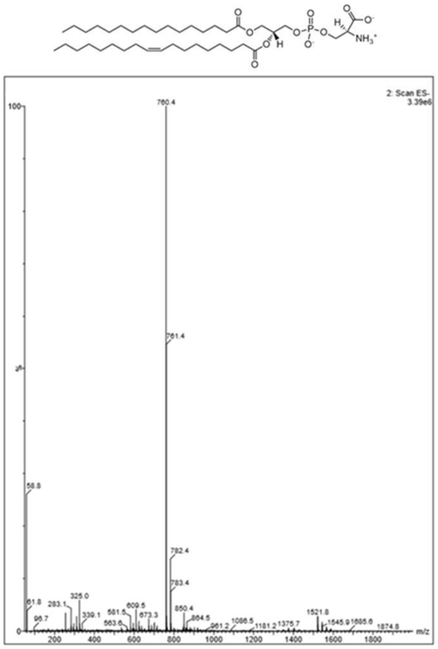
2: Scan ES-
3.39e6
100
%
0
760.4
761.4
58.8
782.4
783.4
61.8
96.7
283.1
325.0
339.1
563.6
581.5
609.5
673.3
850.4
864.5
961.2
1086.5
1181.2
1375.7
1521.8
1545.9
1685.6
1874.8
200
400
600
800
1000
1200
1400
1600
1800
m/z

k

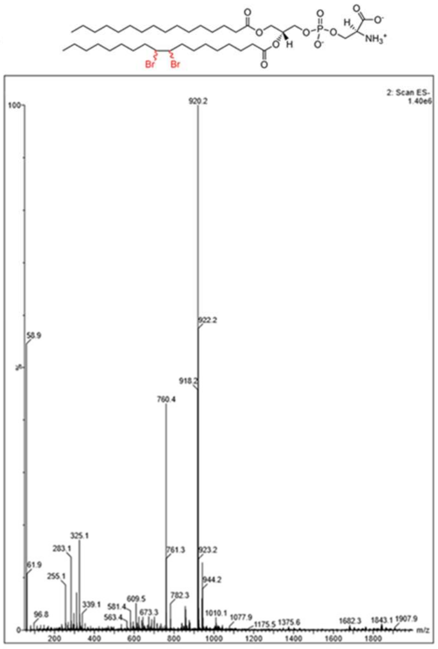
Br
Br
2: Scan ES-
1.40e6
100
%
0
920.2
922.2
918.2
760.4
58.9
325.1
283.1
255.1
61.9
96.8
339.1
563.4
581.4
609.5
673.3
761.3
782.3
923.2
944.2
1010.1
1077.9
1175.5
1375.6
1682.3
1843.1
1907.9
200
400
600
800
1000
1200
1400
1600
1800
m/z

l

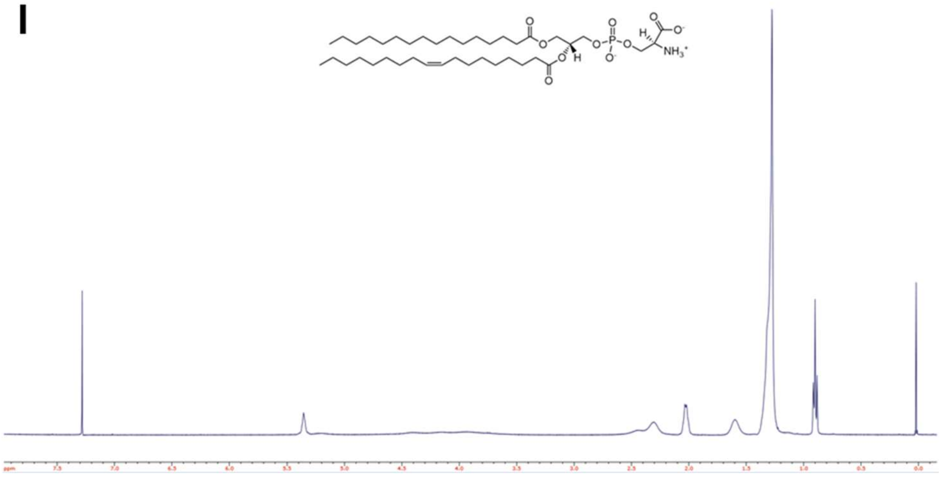

m

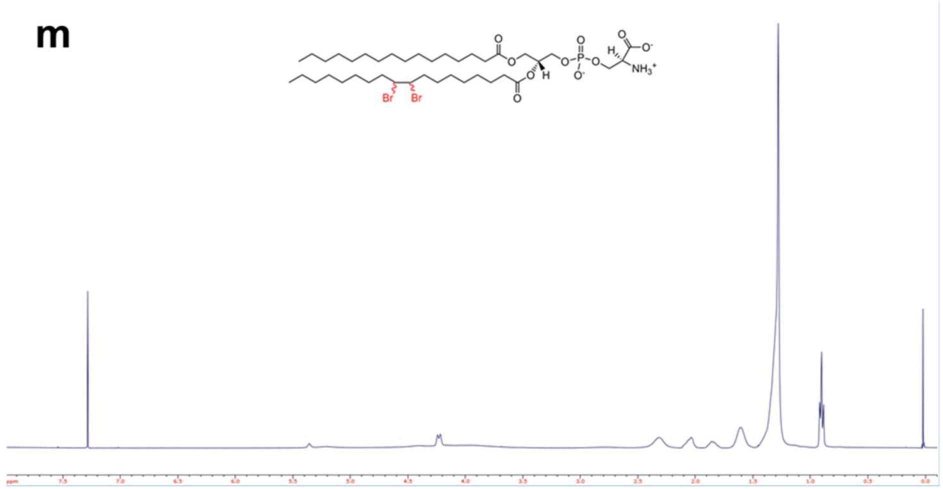

n

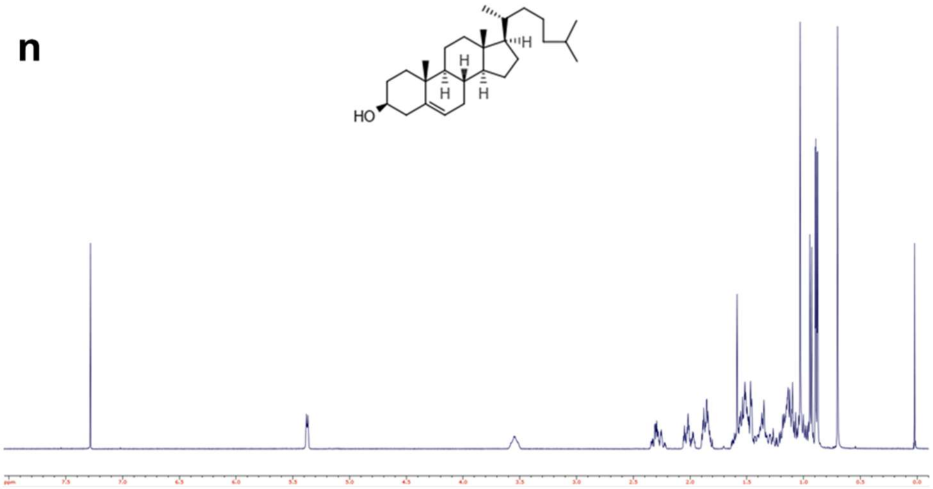

o

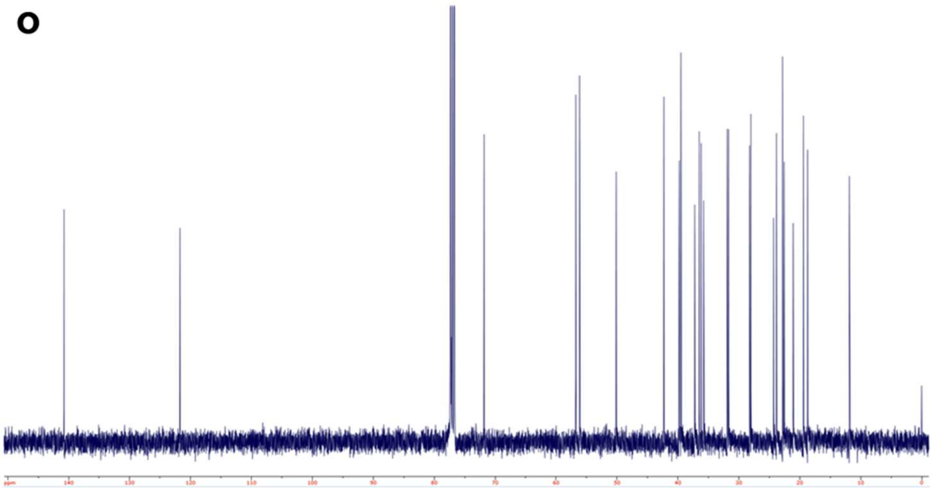

p

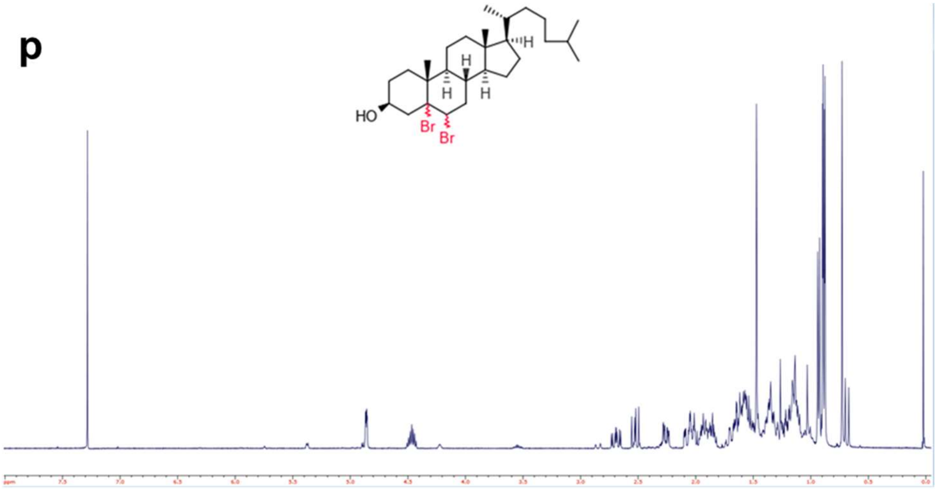

q

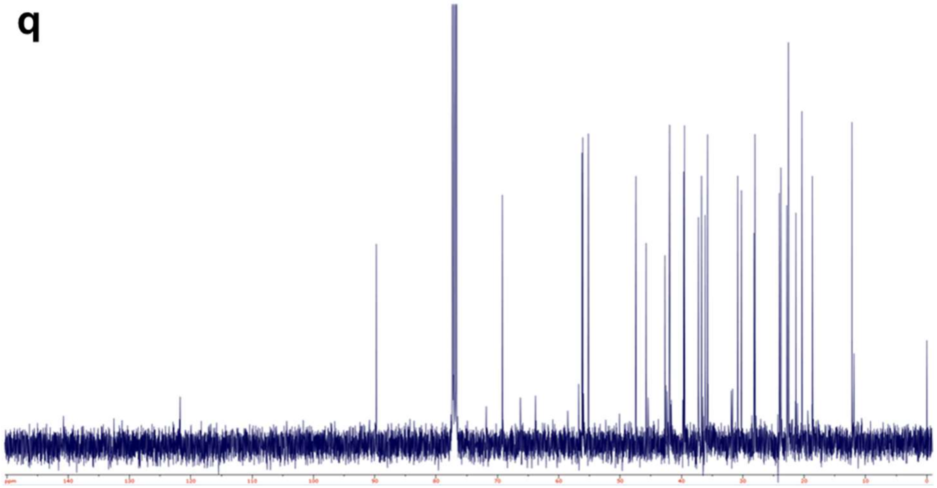

Figure S1. Nuclear magnetic resonance (NMR) and electrospray ionization mass spectrometry (ESI-MS) of brominated lipids. a) ESI-MS spectra of SDPC and b) SDPC-Br. Partially brominated chemical structure is shown to depict the average level of bromination from the mixture of products. c) 1H and d) 13C NMR spectra of SDPC. e) 1H and f) 13C NMR spectra of SDPC-Br. Partially brominated chemical structure is shown to depict the average level of bromination from the mixture of products. g) ESI-MS spectra of PIP_2 and h) PIP_2-Br. i) 1H NMR spectrum of PIP_2-Br. j) ESI-MS of POPS and k) POPS-Br. l) 1H NMR spectrum of POPS. m) 1H NMR spectrum of POPS-Br. n) 1H and o) 13C NMR spectra of CHOL. p) 1H and q) 13C NMR spectra of CHOL-Br.

Lipid	Bromine atoms per molecule
SDPC-Br	7.0
CHOL-Br	1.7
POPS-Br	1.6
PIP_2-Br	2.0

Table S1. Average number of bromine atoms per molecules, calculated from mass spectrometry (SDPC-Br, POPS-Br, and PIP_2-Br) and NMR (Chol-Br) data.

Composition Number	Composition	Components
1	58.2 : 18 : 17.5 : 6.3	SDPC : CHOL : POPS : PIP_2
2	62.1 : 18.7 : 19.2 : 0	SDPC : CHOL : POPS : PIP_2
3	58.2 : 18 : 17.5 : 6.3	POPC : CHOL : POPS : PIP_2
4	71 : 0 : 21.3 : 7.7	SDPC : CHOL : POPS : PIP_2
5	63.9 : 10 : 19.2 : 6.9	SDPC : CHOL : POPS : PIP_2
6	56.8 : 20 : 17.1 : 6.1	SDPC : CHOL : POPS : PIP_2
7	53.2 : 30 : 16 : 5.8	SDPC : CHOL : POPS : PIP_2
8	42.6 : 40 : 12.8 : 4.6	SDPC : CHOL : POPS : PIP_2
9	29.1 : 50 : 8.8 : 3.1	SDPC : CHOL : POPS : PIP_2
10	58.2 : 18 : 17.5 : 6.3	SDPC-Br : CHOL : POPS : PIP_2
11	58.2 : 18 : 17.5 : 6.3	SDPC : CHOL-Br : POPS : PIP_2
12	58.2 : 18 : 17.5 : 6.3	SDPC : CHOL : POPS-Br : PIP_2
13	58.2 : 18 : 17.5 : 6.3	SDPC : CHOL : POPS : PIP_2-Br
14	58.2 : 18 : 17.5 : 6.3	SDPC : CHOL-I : POPS : PIP_2
15	29.1 : 29.1 : 18 : 17.5 : 6.3	SDPC-Br : SDPC : CHOL : POPS : PIP_2
16	14.6 : 43.6 : 18 : 17.5 : 6.3	SDPC-Br : SDPC : CHOL : POPS : PIP_2
17	26 : 22 : 32 : 20	SDPC : CHOL : POPS : PIP_2

Table S2. Lipid mixtures used in membrane remodeling assays. All amounts are mole percentages.

2. Simulation Analysis

2.1 Leaflet Assignment

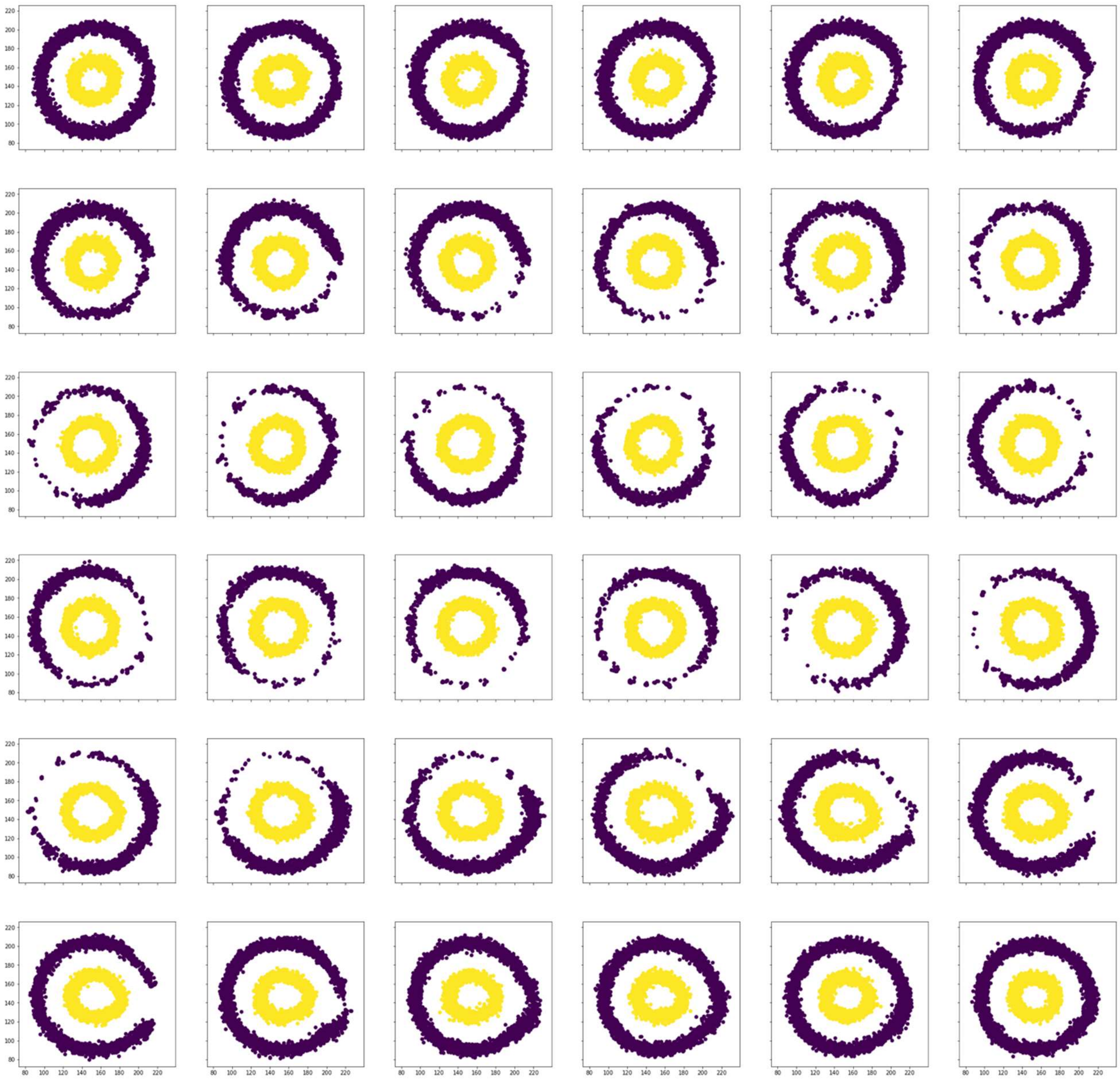

Figure S2. Assignment of lipids to leaflets. Cartesian (x, y) coordinates of SDPC phosphate beads from all analyzed frames of one replicate of WT CHMP1B-IST1 Composition 1 production simulation (SI Section 13.2), sorted into 5 Å bins in z scanning from the bottom of the box (upper left) to the top of the box (lower right), going through subplots left to right in columns and then down in rows. Each SDPC is colored according to the assigned leaflet, dark spots are outer leaflet and yellow spots are inner leaflet. The applied clustering (see Methods) produces cleanly separated leaflets. Scanning through z shows partial exclusion of lipid phosphates spiraling along the helix corresponding to repeats of CHMP1B F9 and F13, and an unperturbed outer leaflet away from the protein at the bottom (upper left few panels) and top (bottom right few panels) of the simulation box.

2.2 Cylindrical Coordinates

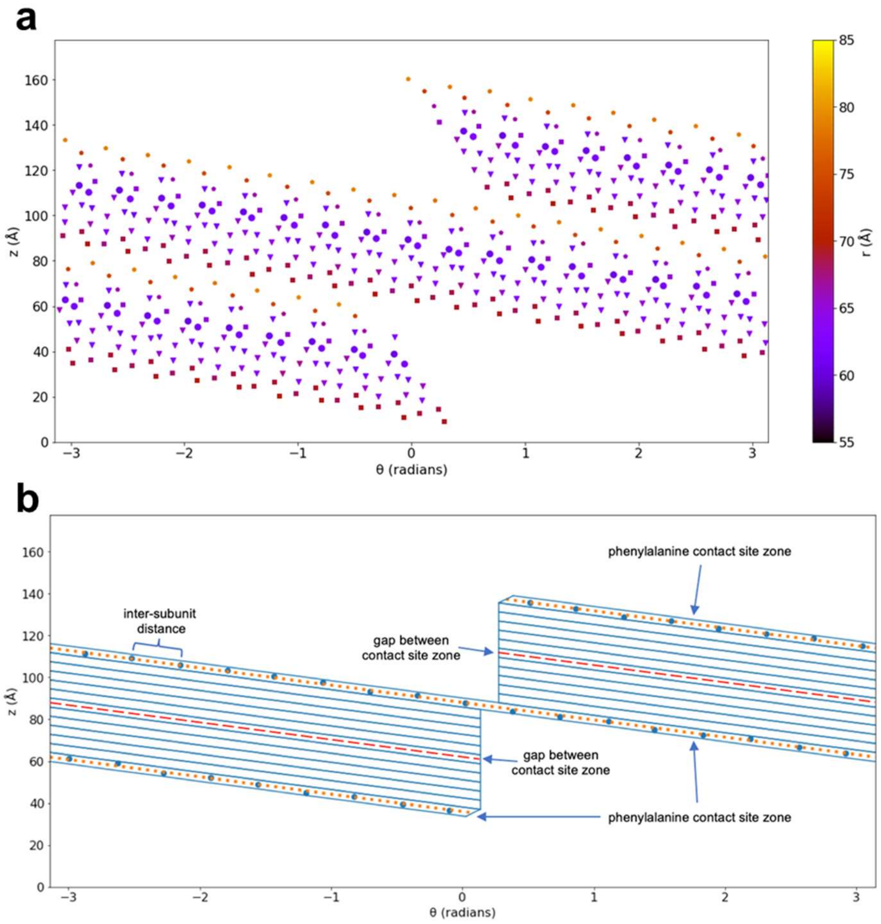

Figure S3. Positions of membrane facing CHMP1B residues represented in cylindrical coordinates and visualization of (θ, z) zones used for analysis. a) The position of the tubule axis in the xy-plane, from which cylindrical coordinates of the protein and lipid are calculated, was updated at each time step to account for subtle motions in the protein coat. The (x_0, y_0) position of axis of tubule was determined by performing least-squares fit on all CHMP1B F9 and F13 positions along the protein coat to place them equidistant from the tubule axis at each time step. Consistent values of r (indicated by color bar) for identical residues from different CHMP1B subunits indicate that the procedure results in well-fit consistent cylindrical coordinates applicable to the lipid tubule. Color variation shows that the F9+F13 contact site sits the most inward toward the tubule central axis, and elsewhere the membrane-facing protein surface shifts further out in r. F9 and F13 in circles; K6, K12, K16, R20, K23, K24, and K27 in triangles; K30, K35, K38, and K87 in squares; and K90, K97, and K104 in pentagons. b) The line fit through mean coordinates of each F9 and F13 pair (large dots) defines the principal axis of each zone (shown as continuous spaces encompassed by solid blue lines). The "phenylalanine contact site" zone is the helical 30 ° wedge centered around the F9 and F13 fit line (dotted orange line), capped as described above (SI 2.3). All additional zones are successive 30 ° slices layered between the continuous phenylalanine contact zone, capped with vertical lines. The "gap between contact site" zone (wedge around dashed red line) is the zone 180 ° opposite the phenylalanine contact site, i.e., the center of the additional 11 zones (see main text Fig. 2).

2.3 Zone Assignment

To quantify variation in lipid composition and structural properties depending on the position relative to certain features of the protein coat, primarily the helical stripe formed by CHMP1B F9 and F13, we defined zones in (θ, z) space based on the protein coordinates. Within these zones we calculated distributions and averages of lipid and bilayer properties, for comparison to other zones (see Fig. S3b). The primary zone consisted of a 30 ° wedge centered around

the helix formed by the positions of CHMP1B F9 and F13. For each frame, the cylindrical coordinates of these residues were gathered, and we used the midpoint of each pair from the 34 subunits to fit a line in (θ, z) space. The "phenylalanine contact site" zone was defined to fit the 30 ° wedge centered around this line. The zone was extended at the ends an extra 1.2 nm (1/2 the length between pairs of F9 and F13 on adjacent CHMP1B subunits), due to the observed continuity between subunits of the membrane surface defect. The zone was then capped with lines perpendicular to the central phenylalanine-fit line in (θ, z) coordinate space at each end. All additional zones were defined as successive 30 ° helical slices following the same slope to cover the entire protein-contacting surface bound in between the helix formed by F9 and F13 (compare Fig. S3b with Fig. S3a). The zone 180 ° opposite the F9 and F13 stripe is referred to as the "gap between contact site" zone. Properties such as local composition and lipid shapes were then calculated for the various zones over time and compared in order to identify local perturbations that correspond with specific residues and features along the CHMP1B membrane-facing surface.

2.4 Leaflet and Bilayer Thicknesses

On a per zone basis, we calculated the locations of the bilayer midplane and planes for the outer and inner leaflet glycerols as follows (ED Fig. 2c). For the midplane calculations the normalized radial densities of all hydrophobic bead types of the inner and outer leaflets (SI Section 2.1) were gathered. Next, the point of intersection along the radius r where the inner leaflet hydrophobic density matched that of the outer leaflet was used to define the midplane. The mean radial coordinate of the glycerol beads in the inner and outer leaflets across the production run within the zone were calculated and values used as the inner and outer leaflet glycerol planes per zone. The per zone leaflet thicknesses were calculated as the differences between the glycerol plane locations and the midplane and were then averaged across the twelve zones and across all simulations to obtain a mean thickness for the entire membrane surface for comparison to cryo-EM. We chose to use the glycerols to calculate leaflet thicknesses, as opposed to other lipid beads, because thicknesses based on the glycerols have been found to better correspond with experimentally derived leaflet thicknesses in with other work.[5]

2.5 Diffusion Coefficients

We calculated lipid diffusion coefficients to determine the impact of the tubule curvature and the protein coat on lipid dynamics. For each individual lipid, the mean squared displacement (MSD) was calculated using unwrapped cylindrical coordinates. On a cylindrical surface, the minimum distance between two points is a helix with arc length $s = \Delta\theta\sqrt{\langle r\rangle^2 + (\Delta z/\Delta\theta)^2}$, where $\Delta\theta$ is the unwrapped change in angular coordinate, Δz is the change in z coordinate, and r is the cylindrical radius. The MSD as a function of time was calculated using increments of 12 ns, on the range spanning from 12 ns to 1.2 μs. Windows were sample across the full 2.4 μs of production data. Since the radial position, r, of each lipid fluctuates, we calculated s using the time-series average value $\langle r\rangle$ computed over the appropriate time window. Previous work has shown that apparent MSDs computed on curved geometries depends on which lipid bead is used for the calculation.[3] We therefore calculated MSDs using the second glycerol beads, as a central element of each phospholipid. Individual MSD time series were averaged over all lipids of a given type for a given leaflet, then a least-squares fit over the sampling timespan from 600 to 1200 ns was used to calculate the diffusion coefficient D (ED Fig. 10).

2.6 Backmapping Procedure

Standard Martini backmap protocols were followed to generate all-atom configurations from CG simulation frames. All-atom CHARMM36 parameters were used for lipids and protein. A CHARMM36 topology and CG mapping file were constructed for di-oleoyl PIP_2, using pre-existing files for 1-palmitoyl-2-oleoyl PIP_2. Images presented in the text are taken after the two rounds of minimization during backmapping: without and then with non-bonded forces turned on, to get all-atom representations that most directly correspond to CG configurations sampled during production. A custom shell script was used in between the first and second rounds of minimization to manually move apart pairs of atoms within 0.5 Å of each other to avoid clashes that would cause the second round of minimization to crash.

3. Cryo-EM Imaging and Data Processing

Process	Parameter(s)	Numerical Value
Extraction	Particle Box Size	480 px
2D Classification	Tube Diameter	250 Å
Initial 3D Auto-Refine (Helical tab)	Inner Tube Diameter / Outer Tube Diameter / asu / initial twist / initial rise	25 Å / 260 Å / 17 asu / 21.1 deg / 3.18 Å
3D classification (17 asu, Optimization tab)	Number of Classes / Regularization Parameter / Mask Diameter	10 / 8 / 350 Å
3D classification (17 asu, Helical tab)	Inner Tube / Outer Tube Diameter / asu / initial twist / initial rise	25 Å / 260 Å / 17 asu / 21.1 deg / 3.18 Å
3D classification (18 asu, Optimization tab)	Number of Classes / Regularization Parameter / Mask Diameter	10 / 8 / 350 Å
3D classification (18 asu, Helical tab)	Inner Tube / Outer Tube Diameter / asu / initial twist / initial rise	25 Å / 260 Å / 18 asu / 20.0 deg / 2.94 Å
3D autorefinement (17asu, Helical tab)	Mask Diameter / Inner Tube Diameter / Outer Tube Diameter / asu / initial twist / initial rise	350 Å / 20 Å / 260 Å / 17 asu / 20.8 deg / 3.18 Å
3D autorefinement (18 asu, Helical tab)	Mask Diameter / Inner Tube Diameter / Outer Tube Diameter / asu / initial twist / initial rise	350 Å / 20 Å / 260 Å / 18 asu / 20 deg / 3 Å
CTF refinement	Minimum resolution for fits / Range for defocus fit	30 Å / 2000 Å

Table S3. RELION data processing parameters for Krios data sets.

Cryo-EM data collection, refinement and validation statistics

	NoBr 17asu (EMD-28700)	NoBr 18asu (EMD-28701)	CHOL-Br 17asu (EMD-28699)	CHOL-Br 18asu (EMD-28722)
**Data collection and processing**				
Magnification	105,000			
Voltage (kV)	300			
Electron exposure (e–/Å^2)	67			
Defocus range (μm)	-0.6 to -2.0			
Pixel size (Å)	0.835		0.822	
Micrographs (no.)	5,684		1,855	
Symmetry imposed	C1, Helical			
Rise (Å)	3.07	2.99	3.08	3.00
Twist (deg.)	-20.81	19.99	-20.82	20.00
Initial particle images (no.)	279,180		53,787	
Final particle images (no.)	26,764	4,103	12,904	12,868
Map resolution (Å)	3.1	3.7	3.8	4.3
FSC threshold	0.143			

CHOL-I 17asu (EMD-28702)	CHOL-I 18asu (EMD-28703)	PIP2-Br 17asu (EMD-28697)	PIP2-Br 18asu (EMD-28698)	POPS-Br 17asu (EMD-28695)	POPS-Br 18asu (EMD-28696)
105,000					
300					
67					
-0.6 to -2.0					
0.822		0.835		0.822	
3,186		2,116		7,806	
C1, Helical					
3.05	2.93	3.06	3.00	3.09	3.00
-20.82	19.97	-20.81	20.00	-20.81	19.99
283,543		170,300		202,716	
63,616	57,773	17,971	6,698	45,351	16,746
3.2	3.1	3.2	3.5	2.8	3.1
0.143					

SDPC-Br 17asu (EMD-27991)	SDPC-Br 18asu (EMD-28694)	50% SDPC-Br 17asu (EMD-28706)	50% SDPC-Br 18asu (EMD-28707)	25% SDPC-Br 17asu (EMD-28704)	25% SDPC-Br 18asu (EMD-28705)
105,000					
300					
67					
-0.6 to -2.0					
0.822		0.822		0.822	
2,875		3,891		2,931	
C1, Helical					
3.09	3.02	3.05	2.97	3.07	2.99
-20.81	20.00	-20.81	19.99	-20.83	20.00
188,013		256,608		285,067	
30,752	49,713	41,516	49,975	46,287	66,567
2.9	3.0	2.9	3.0	3.1	2.9
0.143					

SDPC-Br F9A F13A 17asu (EMD-28713)	SDPC-Br F9A F13A 18asu (EMD-28714)	SDPC-Br F9E F13E 17asu (EMD-28715)	SDPC-Br F9E F13E 18asu (EMD-28716)	SDPC-Br F9L F13L 17asu (EMD-28717)	SDPC-Br F9L F13L 18asu (EMD-28718)
105,000					
300					
67					
-0.6 to -2.0					
0.822		0.822		0.822	
1,715		1,420		2,188	
C1, Helical					
3.05	2.95	3.10	2.98	3.10	3.00
-20.81	19.99	-20.79	20.01	-20.80	20.01
251,839		65,690		186,495	
66,916	51,093	21,747	6,994	44,673	16,539
2.9	3.0	3.4	3.5	3.2	2.9
0.143					

Table S4. Krios data collection and processing statistics.

Cryo-EM data collection, refinement and validation statistics

0% CHOL 17asu (EMD-28708)	10% CHOL 17asu (EMD-28709)	20% CHOL 17asu (EMD-28710)	30% CHOL 17asu (EMD-28711)	40% CHOL 17asu (EMD-28712)	POPC Mixture 34asu (EMD-28719)
28,000				36,000	
61					
-0.6 to 2.0					
1.14				1.43	
615	653	816	492	481	502
C1, Helical					
3.14	3.15	3.20	3.15	3.13	1.56
-20.82	-20.91	-20.81	-20.92	-20.81	10.52
47,415	54,720	33,006	22,959	13,974	20.545
2,772	3,373	2,505	1,193	3,677	2,864
4.1	4.0	4.9	4.4	4.7	9.5
0.143					

Table S5. Arctica data collection and processing statistics.

4. Analysis of leaflet compositions

Lipid	Inner Leaflet (mol%)	Outer Leaflet (mol%)
CHOL-Br	28 ± 4	20 ± 6
PIP_2-Br	17 ± 2	25 ± 5
POPS-Br	36 ± 6	33 ± 9
SDPC-Br	19 ± 2	22 ± 4

Table S6. Radially averaged leaflet lipid compositions. The uncertainties are the standard deviations between independent half maps.

5. Cryo-EM Data for all Brominated Lipid Samples

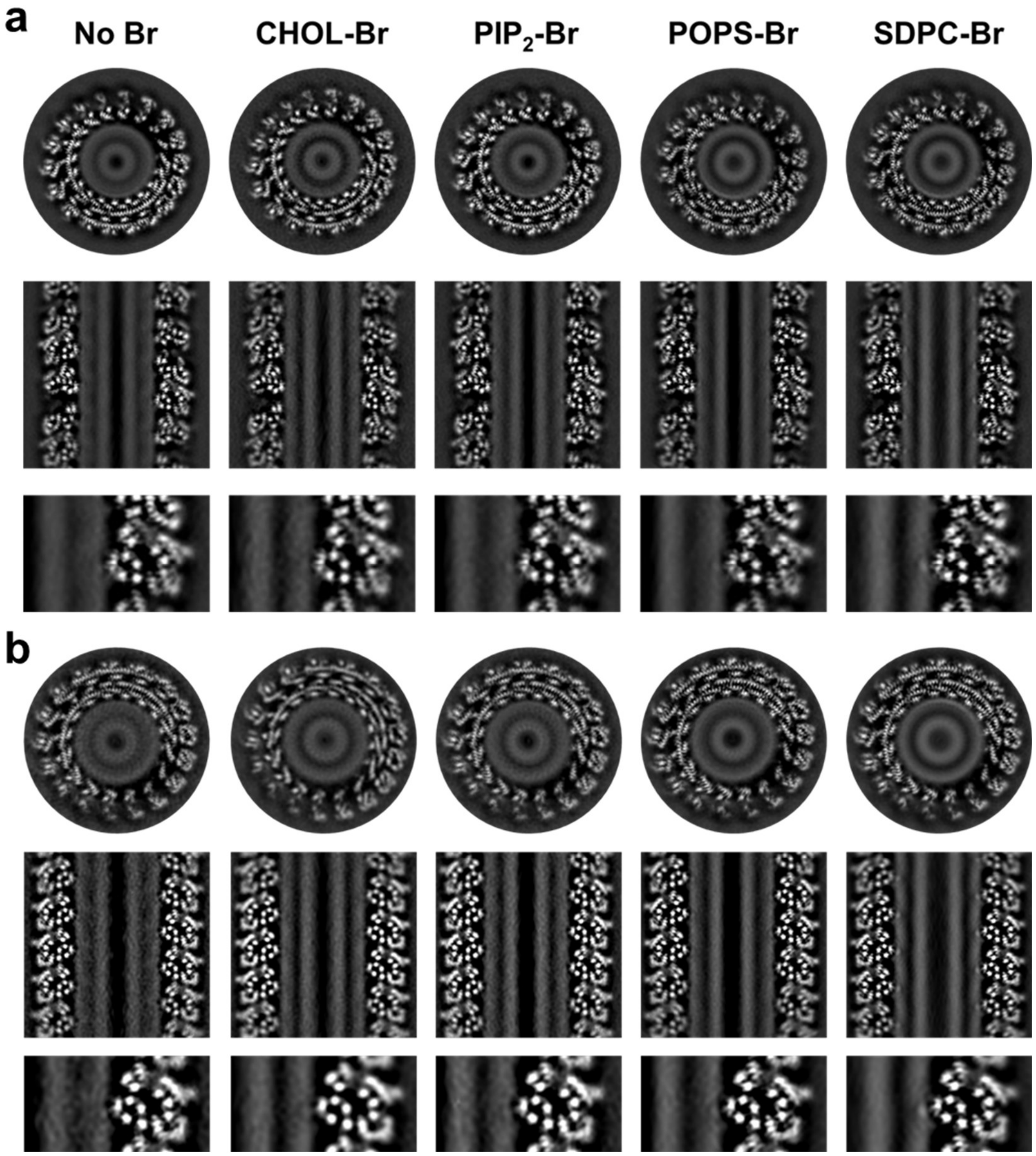

Figure S4. Horizontal and vertical slices from all reconstructions. a) Horizontal (top) and vertical (middle) slices through the cryo-EM reconstructions of 17 subunit-per-turn CHMP1B/IST1 filaments with and without brominated lipids. The bottom panels show an enlarged view of the membrane-protein contact sites. b) Horizontal (top) and vertical (middle) slices through cryo-EM densities for 18-subunit-per-turn structures with and without bromolipids.

As shown in Fig. S4a, each of the brominated lipids resulted in cryo-EM reconstructions with different patterns of intensity in the lipid bilayer. For example, CHOL-Br appears to uniformly increase the intensity of the bilayer, while the phospholipids tend to segregate asymmetrically. The phospholipids increase the intensity of each leaflet of the bilayer by different amounts and accumulate to variable extents where CHMP1B contacts the bilayer. These qualitative observations are quantified in Fig. 3 and Fig. 5.

We also generated high resolution cryo-EM reconstructions of 18 subunit per turn, right-handed filaments, as described previously.[4] They display the same features and trends as the 17 subunit per turn, left-handed filaments. See slices through the reconstructions in Fig. S4b.

6. Negative stain TEM

Negative stain EM was routinely performed to assess the formation and morphology of vesicles and membrane-bound CHMP1B/IST1 filaments. Vesicles and filaments formed with bromolipids were indistinguishable from those formed with unbrominated lipids. ED Fig. 3 shows a representative micrograph of CHMP1B/IST1 filaments with lipid composition 1 (Table S6), and Fig. S5 shows representative micrographs of CHMP1B/IST1 with lipid compositions 11 (a) and K16E/R20E CHMP1B and IST1 with lipid composition 1 (b).

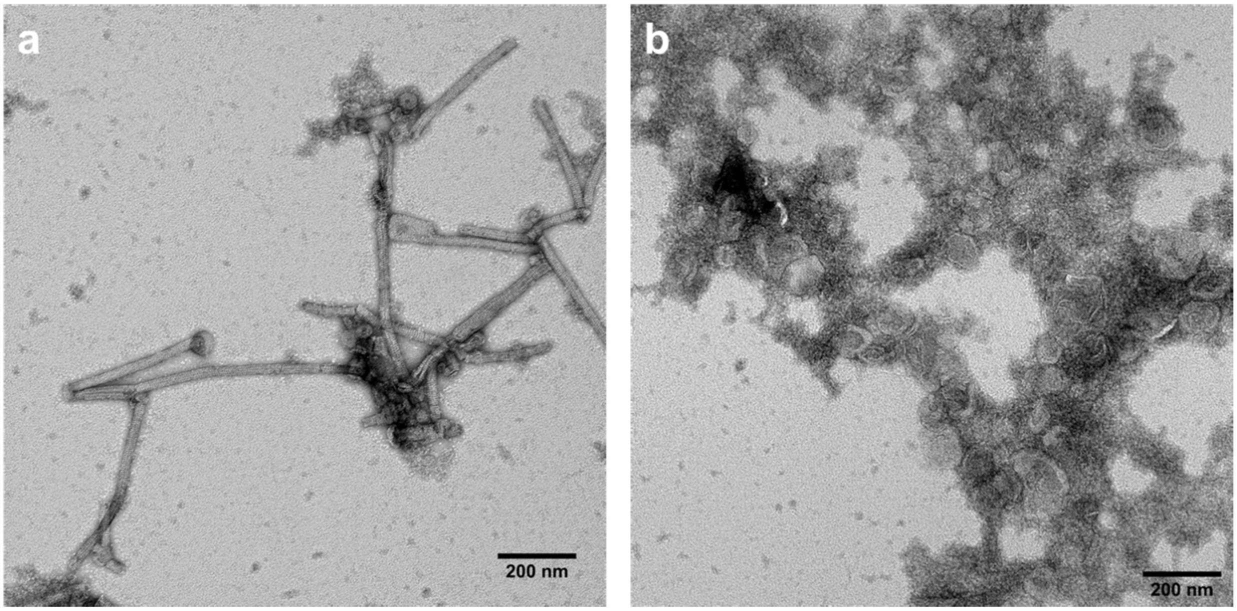

Figure S5. Negative stain TEM micrographs of CHMP1B/IST1 filaments. a) Wild type CHMP1B and IST1 with vesicles made from lipid composition 11 (Table S6). Filaments look identical to those made with lipid composition 1 (ED Fig. 3a). b) K16E/R20E CHMP1B and IST1 mixed with vesicles made from lipid composition 1 (Table S6). The double mutant protein does not bind to or remodel vesicles. Micrographs are representative of approximately 10 collected for each sample.

7. Effects of data collection and analysis parameters

We examined whether data collection and analysis parameters had any effect on the final 3D reconstructions by examining the radial profiles. As shown in Fig. S6 applying low-pass filters, helical symmetry, and normalization to CHMP1B helix α1 had no meaningful effects on the bilayer or protein density.

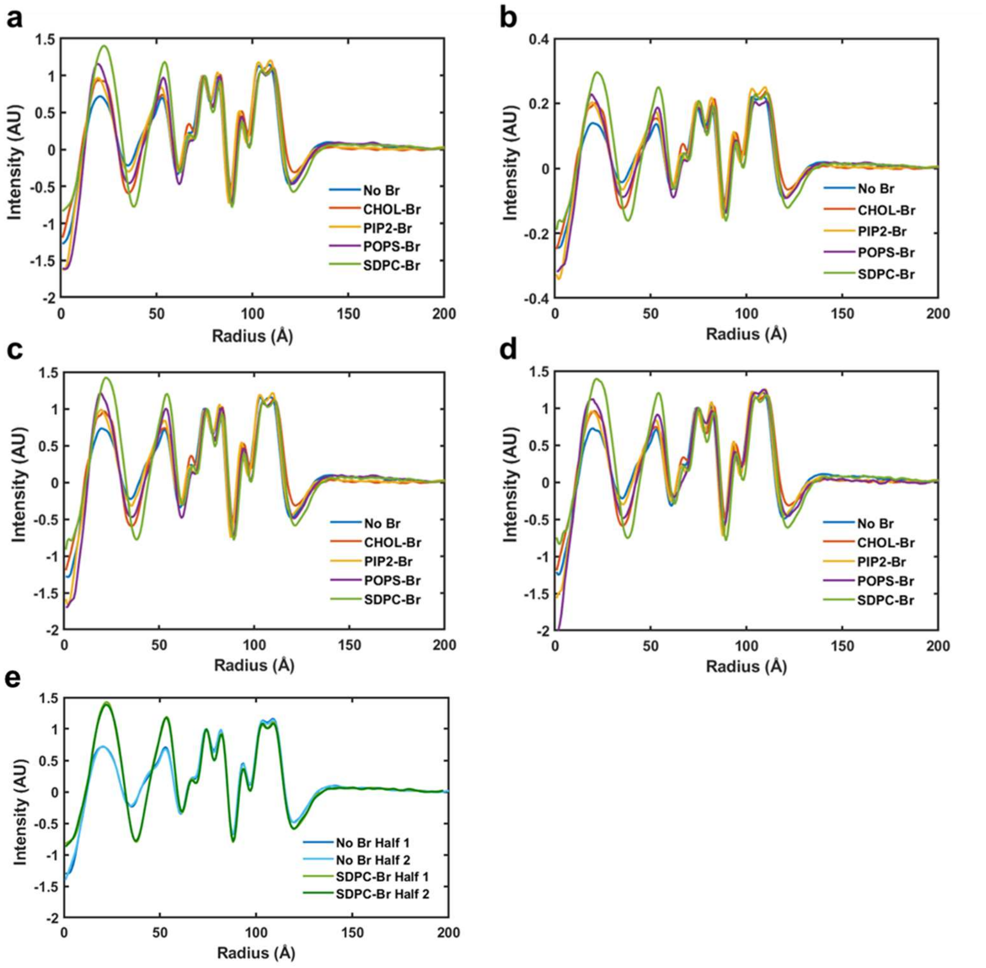

Figure S6. Effects of data processing on electron scattering profiles. a) Normalized electron scattering profiles with helical symmetry and 6 Å low pass filter applied. b) Unnormalized electron scattering profiles with helical symmetry and 6 Å low pass filter applied. c) Normalized electron scattering profiles with helical symmetry applied and no low pass filter applied. d) Normalized electron scattering profiles without helical symmetry or low pass filter applied. All electron scattering profiles are from radial averages of Z-projected density maps. e) Comparison of half maps for the reconstructions with unbrominated lipids and SDPC-Br (Table S6, lipid compositions 1 and 10). We took advantage of these half maps to estimate uncertainty in our analyses as the standard deviation of the measured quantity between the two half maps.

8. Effects of varying lipid composition

We systematically varied the lipid composition of the vesicles in order to examine the effects on remodeling by CHMP1B and IST1. Vesicles and filaments formed with bromolipids were indistinguishable from those formed with unbrominated lipids (see ED Fig. 3a and Fig. S5a). However, changing the lipid composition of the vesicles in other ways did produce morphological changes in the membrane-bound CHMP1B/IST1 filaments. Substituting POPC for SDPC (composition 3) caused the resulting filaments to be significantly wider than those formed from composition 1 (see ED Fig. 3c,e,g,k). The mole percent of cholesterol was also varied between 0 and 50, while keeping the ratio of the other three components constant (Table S6, lipid compositions 4 – 9). We repeated the previously described membrane remodeling assay with vesicles made from each of these lipid compositions and visualized them with negative stain EM. As shown in ED Fig. 3a and 9d, CHMP1B and IST1 remodel vesicles from compositions 4 – 8 (0 – 40% cholesterol) into membrane-bound filaments that are indistinguishable from those formed from lipid composition 1 (Table S6, 18% cholesterol). However, at 50% CHOL, CHMP1B and IST1 bound to vesicles but could not remodel them into thin nanotubes. Finally, removing the PIP_2 from the lipid mixture (Table S6, lipid composition 2) abolished protein binding to the vesicles (ED Fig. 3i and 3m).

SDPC is known to reduce the bending rigidity of lipid bilayers, while cholesterol is known to increase the bending rigidity of fluid phase bilayers. Both replacing SDPC by the less flexible POPC and increasing the cholesterol concentration causes the filaments to become wider. These observations are consistent with a simple mechanical model in which CHMP1B and IST1 apply force to the bilayer, whose final diameter depends on the bending rigidity and spontaneous curvature of the bilayer. As SDPC and cholesterol likely change both the bending rigidity and spontaneous curvature of the bilayer, more work is needed to disentangle these effects.

The failure of tubule formation to occur after removing PIP_2 from the lipid mixture highlights the importance of electrostatic interactions between CHMP1B and the bilayer. Similarly, mutation of K16 and R20 to Glu abolishes CHMP1B binding to bilayers containing PIP_2 (Fig. S5b).

Finally, cholesterol is significantly different structurally than the phospholipids in the lipid mixture and has lower electron density. We exploited this difference to confirm that cholesterol is overall enriched in the inner leaflet of the bilayer, consistent with the data from CHOL-Br (Table S7). We tracked into which leaflet cholesterol partitions by titrating from 0 to 40 mol% cholesterol and quantifying how much each leaflet decreased in electron scattering intensity. The inner leaflet decreases in intensity preferentially up to 30 mol% cholesterol, after which it saturates (ED Fig. 9d).

9. Sub-stoichiometric SDPC-Br

We probed whether SDPC-Br has the same affinity for the CHMP1B-bilayer contact sites as SDPC. We performed a titration by substituting 25 % and 50 % of the SDPC in lipid composition 1 (Table S6) for SDPC-Br, resulting in lipid compositions 16 and 15 (Table S6), respectively. We then created 3D reconstructions of the resulting filaments as previously described and compared the intensities at the protein-bilayer contact sites. If SDPC-Br behaves exactly the same as SDPC, the intensities at the contact sites will be linear with SDPC-Br mol %.

As shown in ED Fig. 4e-g, lipid bilayers in which less than 100 % of the SDPC is brominated display intensities at the contact site that are intermediate between samples with 0 % and 100 % SDPC-Br. The change is not completely linear and shows somewhat higher than expected SDPC-Br enrichment when the SDPC-Br mol fraction is between 0 % and 100 %. This result suggests that SDPC-Br has a slightly higher affinity for the CHMP1B-bilayer contact sites than SDPC or that SDPC-Br is more enriched in the lipid bilayer within the protein coat than SDPC. Lipid bilayer vesicles are often seen protruding from the ends of filaments (see ED Fig. 3a,f,j and Fig. S5a). This scenario provides the opportunity for some lipids to be enriched in the highly curved lipid bilayer nanotube inside the protein coat, while others are enriched in the less curved vesicle. We note that the differences between SDPC and SDPC-Br are likely small as the contact sites do not saturate at lower SDPC-Br mole fractions.

10. CHMP1B Phe double mutants

We investigated the chemical and mechanical effects of CHMP1B F9 and F13 on the lipid bilayer by creating a series of double mutants in which the Phe residues were replaced by residues with varying size and hydrophobicity. Surprisingly, all of the double mutants tested bound to vesicles and remodeling them into high curvature nanotubes. We performed cryo-EM imaging and reconstruction on each of these CHMP1B constructs with IST1 and lipid composition 10, (Table S6) which contains SDPC-Br. While the protein coat was identical to the wild type CHMP1B/IST1 coat (except for the mutated residues), the underlying lipid bilayer appeared quite different in each case. The results are shown in ED Fig. 5. Counterintuitively, the smallest side chain, Ala, resulted in deep elastic deformations of the bilayer outer leaflet. The larger, charged Glu side chains had a similar effect on the bilayer. Interestingly, in both cases, no accumulation of SDPC-Br at the contact sites was observed. As discussed in the main text, we hypothesize that the SDPC-Br sn-2 tail does not interact with the small, less hydrophobic Ala and charged/polar Glu side chains, so it does not accumulate there. Without the formation of the hydrophobic defect at the bilayer surface characterized by a stripe of back-flipped lipid tails, the outer leaflet instead elastically deforms due to the presence of the charged CHMP1B helix α1. Lipid headgroup density is less depleted than for the wild type CHMP1B. Consistent with this model, substitution of the Phes for Leu, which is intermediate between Ala and Phe in both size and hydrophobicity, eliminates the elastic deformation in the outer leaflet and partially restores the SDPC-Br accumulation at the contact site. We hypothesize that such large changes in the structure of the bilayer will have consequences for the energy required to bend the bilayer and the energy necessary to cross the energy barrier to fission. In the absence of a cell-based assay for membrane remodeling by CHMP1B and IST1, these hypotheses remain difficult to test *in vivo*.

11. 19-Iodocholesterol

We also evaluated the 19-iodo derivative of cholesterol (CHOL-I), which is commercially available from Sigma-Aldrich. Examining the cryo-EM reconstructions, samples with CHOL-I appeared qualitatively similar to those containing CHOL-Br, albeit with less added intensity (Fig. S7), as expected based on the electron atomic scattering factors for two bromine atoms versus one iodine atom. As the iodine atom is in a different location than the bromine atoms and should be less perturbative than two bromine atoms, the similar behavior compared to both CHOL and CHOL-Br supports the idea that halogenation of cholesterol is minimally perturbative to its behavior in lipid bilayers.

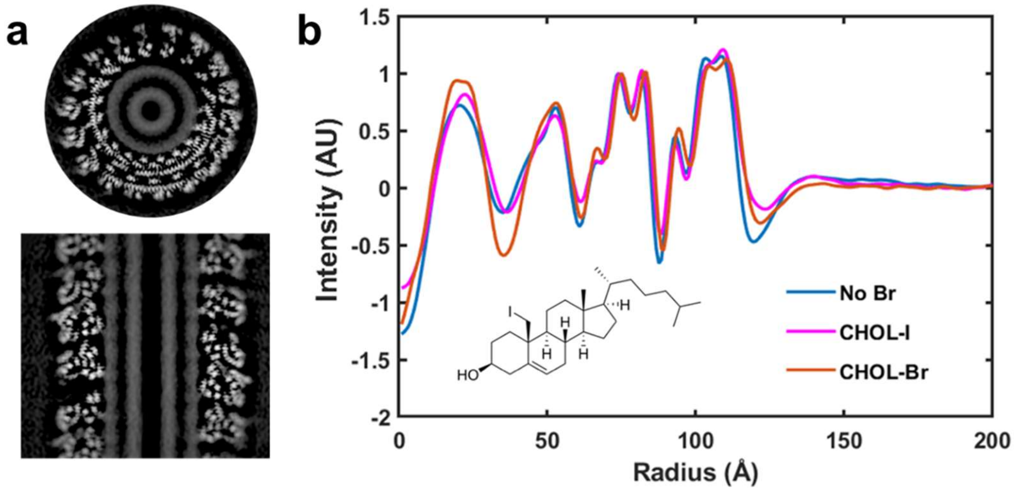

Figure S7. Reconstructions and radial profiles with CHOL-I and comparison to CHOL-Br. a) Horizontal (top) and vertical (bottom) slice through cryo-EM reconstruction with CHOL-I. b) Comparison of radial profiles of cryo-EM reconstructions from samples with no brominated lipids, CHOL-I, and CHOL-Br.

12. Pressure-area isotherms of lipids and bromolipids

We next examined the effects of bromination on the behavior of brominated lipids within a lipid monolayer at the air-water interface.[1] We compared the monolayer behavior of the lipids to the unsaturated lipids from which they were prepared and to an analogous, representative saturated lipid (DSPC). As shown in ED Fig. 4h-i, the isotherms for SDPC-Br and POPS-Br are similar to those for SDPC and POPS, respectively. For both brominated lipids, the mean molecular areas (MMAs) are close to those of the analogous unsaturated lipids at 32 mN/m, the surface pressure that approximates the packing in a lipid bilayer.[2] Furthermore, both brominated lipids have compressibilities that are characteristic of lipids in the fluid phase at room temperature and similar to those of the analogous unsaturated lipids. The compressibility of the lipid monolayer is equal to the negative slope of the isotherm at a given surface pressure (in this case, 32 mN/m). In both cases, the brominated lipids are slightly less compressible than their unsaturated analogs. As a comparison, a representative saturated phospholipid, DSPC, is show in ED Fig. 4h. The isotherm of DSPC displays behavior typical of a saturated phospholipid with a transition temperature above room temperature. Its MMA and compressibility are much lower than those of the unsaturated and brominated lipids. See Moss et al for more examples of pressure-area isotherms for a variety of lipid structures[1]. The pressure-area isotherms for sterols are shown in ED Fig. 4i. The pressure-area isotherms for CHOL-Br and CHOL-I are similar to that of CHOL. CHOL-Br has a slightly lower MMA at 32 mN/m than CHOL, while CHOL-I has a slightly larger MMA. The compressibilities for all three sterols are similar. Small differences in MMAs also reflect the uncertainty in the lipid stock concentrations. Overall, these data demonstrate that in monolayers, the brominated lipids behave in a similar manner as the unsaturated lipids from which they are synthesized.

13. CHMP1B-IST1 Composition 1 Tubule Simulation Setup and Parameters

Protein-membrane tubule simulations consisted of three main steps described in detail below: tubule self-assembly (14.1), then leaflet equilibration (14.2), and then production (14.3). Main run parameters are described in the Methods sections.

13.1 Tubule Self-Assembly

The initialization protocol was developed to promote rapid tubule assembly by initially placing randomly oriented lipids within the entire cylindrical space encompassed by the protein coat. A set of example input file, outputs files, and the in-house scripts written for lipid placement are included in the supplement, and instructions for their use are written at the end of this section, following the general description of the procedure and the subsequent solvation and standard MD for spontaneous assembly.

An even grid of positions spaced 4 Å in each direction was generated, containing points within a 5.75 nm radius cylinder centered in the box along the z-axis. This radius was chosen to avoid placing lipids so near the protein that they would immediately insert into the protein coat rather than joining the tubule. A shell script was then used to iterate through the list of grid positions, using the Gromacs insert-molecules function to add lipids one at a time at each grid position until the desired composition and packing were reached. The order of the list of grid positions was randomized for every new replicate, such that individual replicates started with different initial placements for the lipids. This was done to improve sampling of lipid localization and overcome the limits in lipid diffusion in the protein-associated outer leaflet. After lipids were placed, the systems would be solvated and minimized as described in the Methods. Standard MD was then run using a 0.03 ps timestep and semi-isotropic Berendsen barostat. Tubulation was readily achieved within the low hundreds of ns. Each tubulation phase was run for 1.5 µs before moving to leaflet equilibration, described in Section 2.2.

The initial placement of lipids introduces a strong biasing effect toward rapid formation of a tubule, which is desired for efficiency but also brings up concerns about proper equilibration of lipids between leaflets, amount of water within the tubule lumen, and total amount of lipid within the box. The first two concerns are handled with leaflet equilibration, described in SI 2.2. To identify the proper number of lipids, we ran initial tubulation simulations using a range of total lipid counts, shown in ED Fig. 1a-f.

Lipid Placement Example Files and Use

We have provided a set of example input and output files and the python and bash scripts written for the lipid packing protocol. The files are uploaded to Zenodo (https://doi.org/10.5281/zenodo.7232344). Their use requires: a working installation of Gromacs, a Gromacs-formatted .gro coordinate file for the protein complex, Martini .gro coordinate files for each lipid type to be added (available from the Martini website), and basic familiarity with the terminal, shell scripting, Gromacs, and Martini.

Input files:
6tz5_protein_coords.gro – Gromacs coordinate file for Martini representation of the CHMP1B-IST1 complex, centered in the periodic box. Used to make 6tz5_protein_coords.dat, and later for adding lipids using add_lipids.sh.

6tz5_protein_coords.dat – List of protein bead coordinates generated from 6tz5_protein_coords.gro with simple text editing, for input into gen_position_grid.py.

Scripts:
gen_position_grid.py – Generates a space-delimited, three-column list of xyz-coordinates ‘trial_positions_clean.dat’ that will be shuffled and then used as trial positions for inserting lipids into the protein-containing structure file. Can be run from the terminal using ‘python gen_position_grid.py’ in a directory that contains the list of protein coordinates ‘6tz5_protein_coords.dat,’ which is used to prevent saving trial positions that are likely to produce clashes with the protein during lipid insertion.

add_lipids.sh – The main lipidation shell script. Uses a shuffled list of trial coordinates made by gen_position_grid.py to add lipids one by one in random orientations to a structure file that contains the protein. Calls the Gromacs function insert-molecules and requires that model Martini coordinate files for each lipid type (available from the Martini website) are accessible, as well as one subdirectory for each lipid type to be added. Precise details are included in the

script header and instructions below. Can be run from the terminal using 'bash add_lipids.sh' and produces final output structure 'protein_and_lipids.gro.'

Output files:
trial_positions_clean.dat – Ordered list of trial positions for lipid insertions generated by gen_position_grid.py. To be shuffled, using e.g. 'shuf trial_positions_clean.dat > trial_positions_shuf.dat', to create the randomized input list that add_lipids.sh will use to add lipids to the protein structure file.

protein_and_lipids.gro – Final output structure containing protein and lipids, made by add_lipids.sh.

Instructions:
1. Save example files to a working directory. Download and verify proper installation of required software (python, Gromacs). Download Martini model lipid coordinate files and save to a common directory. Here, add_lipids.sh looks for model coordinate files in ~/Documents/Martini. The coordinate file for di-oleoyl PIP_2 'DOP2' can be made by modifying Martini lipid POP2 using standard Martini building block rules for lipid tails.
2. Take the Martini-model Gromacs-format protein coordinate file (example: 6tz5_protein_coords.gro) and reformat to a space-delimited list using a text editor like vim or sed (example: 6tz5_protein_coords.dat).
3. Run 'python gen_position_grid.py' to generate a list of trial coordinates (example: trial_positions_clean.dat). The list of protein coordinates '6tz5_protein_coords.dat' generated in step 2 must be in the working directory for this script to run successfully.
4. Shuffle the trial coordinates using 'shuf trial_positions_clean.dat > trial_positions_shuf.dat' or equivalent to generate a randomized list of coordinates.
5. Prepare the working directory to run the lipidation script 'add_lipids.sh.' The working directory must contain a randomized list of trial coordinates 'trial_positions_shuf.dat.' The working directory must also include one subdirectory for each lipid type to be added, here: add_pupc, add_pops, add_chol, and add_dop2. Copy the protein coordinate file '6tz5_protein_coords.gro' into the 'add_pupc' subdirectory and rename the file 'success.gro.' This will be the file that is modified by 'add_lipids.sh' as lipids are added.
6. Run 'bash add_lipids.sh' to add lipids to the protein coordinate file. The final output 'protein_and_lipids.gro' will be copied from the last lipid subdirectory 'add_dop2' to the working directory after completion. This coordinate file can then be solvated and ionized using standard Gromacs functions before minimizing and beginning MD.

13.2 Tubule Leaflet Equilibration

The dense initial packing of lipids means that, during tubulation, there is insufficient opportunity for phospholipid flip-flop to ensure properly equilibrated lipid densities in the two leaflets. We therefore followed the procedure introduced by Risselada and Marrink, which serves to equilibrate leaflet lipid densities—and, for mixed membranes, leaflet compositions—in simulated vesicles.[3] A repulsive flat-bottomed position restraint potential that acts on phospholipid tails and cholesterol was applied, oriented perpendicular to the tubule axis (along the y axis of the simulation box) at the top of the simulation box. The force constant was 1 kJ/mol*nm^2. This introduces two solvated pores at opposite sides of the tubule, at which phospholipids can exchange between the inner and outer leaflets over time until the preferred equilibrium lipid densities and compositions are reached, after which the pore can be closed for production (SI 2.3). These pores also allow water and ions to move between the now-connected compartments of the tubule lumen and the rest of the bulk solvent in the simulation box, allowing the system to reach preferred densities of each component in these spaces.

To smoothly open the pore and avoid possible bubbling from applying too strong a force from the repulsive potential on a lipid, pores were slowly introduced with 3 short, 25 ns phases using a reduced timestep of 0.02 ps and increasing the radius of the repulsive potential in each phase: 0.5, 1.0, then 1.5 nm. Finally, 9 μs of leaflet equilibration with a 2.0 nm pore radius and a 0.03 ps timestep was run. This total simulation length was chosen based on monitoring of leaflet compositions over time during equilibration (ED Fig. 1 g-h). Pressure coupling was changed to the Parrinello-Rahman semi-isotropic barostat for all phases of leaflet equilibration, and the same barostat was continued for production. Following the 9 μs of 2.0 nm pore equilibration, the 3 short 25 ns phases with reduced 0.02 ps timestep were repeated to close the pore, moving between radii of 1.5, 1.0, and 0.5 nm.

13.3 Production

Following leaflet equilibration, production was run for 3 μs using the same MD parameters. The last 2.4 μs of each production simulation was used for analysis, with a 12 ns frame rate.

14. Experimentally Derived Lipid Composition with WT CHMP1B-IST1 Tubule Simulations

Differential scattering analysis of tubules formed with halogen-labeled lipids revealed a difference in protein-formed tubule composition from the bulk lipid composition, motivating a set of simulations that match the experimentally-derived composition of 26:32:22:20 SDPC:POPS:CHOL:PIP_2 (Table S6, lipid composition 17). We initially attempted to carry out simulations with the same procedures and parameters discussed in SI Section 13 but found the new membranes to be substantially less stable, producing persistent vacuum bubbles in the simulation box. We believe this to be due to the much higher negative charge density in the membrane from the enrichments in POPS and PIP_2.

In an attempt to produce stable tubule systems, many modifications, alone and in combination, to the parameters were tested. These included increasing the applied pressure by the barostat, changing the barostat algorithm, reducing the timestep to 15 fs (timesteps of 20-40 fs are generally recommended for Martini 2.2), and waiting to turn on pressure coupling only once the production phase was started. All of these modifications failed to avoid bubble formation. It was ultimately necessary to maintain an NVT system throughout equilibration and production in order to complete these simulations without bubbling. We therefore proceeded with the modified procedure described below. Constant use of the NVT ensemble introduces a possible mismatch between the density based on the initial fixed volume and the true preferred density of the system at the run conditions. In the ten replicates of the set 1 system (SI Section 13), the average box dimensions at the end of production were 29.81 x 29.81 x 17.75 nm, a 0.6 % change in the x and y dimensions and 1.4 % change in the z dimension from the initial 30 x 30 x 18 nm. So, while the required use of NVT for these simulations is not desirable, and we will seek to develop alternative approaches to stably run these and other compositions with pressure coupling on in future work, the error here is tolerable for this set.

In summary, four main changes were made to the simulation procedure, the first and most important of which is the use of NVT throughout. Second, the initial placement of lipids during setup was changed such that none were placed within the cylindrical region of the repulsive pore-forming potential used for leaflet equilibration, and the simulation could therefore begin directly with leaflet equilibration and the repulsive potential on. Third, the applied radii and force constant of the repulsive potential were changed during the three opening and closing steps of the pore, as were the lengths of each—though the main organization of these phases remained the same. Finally, the length of the leaflet equilibration stage was reduced as was the timestep, and this shorter timestep was also used during production.

After editing the set of grid points where lipids are inserted to remove points within the cylindrical space where the repulsive pore-forming potential is applied for leaflet equilibration, the simulations were initialized with the repulsive potential turned on. This was done to reduce overall simulation time, by removing the tubulation step (as in SI Section 13.1), and to reduce the chance that a lipid might experience a strong repulsive force from the repulsive restraint potential during pore formation.

The simulations were therefore started with the three short equilibration steps to expand the pore: 100 ns at radius 1.5 nm, 50 ns at radius 1.75 nm, and 50 ns at radius 2.0 nm, all using a 0.02 ps timestep and a force constant of 0.5 kJ/mol*nm^2 for the repulsive potential. Simulations then continued to 6 μs of leaflet equilibration using a 0.025 ps timestep and the same conditions for the repulsive potential. This simulation time was found to be sufficient for the three replicates to converge to similar leaflet compositions (Table S10). The pores were then closed with three additional short equilibration phases: 50 ns at radius 1.5 nm with a 0.02 ps timestep, 25 ns at radius 1.0 nm with a 0.02 ps timestep, and 7.5 ns at radius 0.5 nm with a 0.015 ps timestep.

For production, the timestep was increased back up to 0.025 ps and the simulation was run for 3 μs, with results from the last 2.4 μs of production used for analysis and comparison with the original set of simulations.

While the need to maintain NVT throughout for these simulations is a concern, the lipid tubules for this set were—compositional differences aside—structurally and behaviorally very similar to those of the initial set run with proper pressure coupling, suggesting that the slight mismatch in likely preferred density was tolerable for the system. Further, the 6 μs leaflet equilibration phase allowed sufficient time to properly distribute solvent between the two eventually separated compartments of the tubule inner lumen and the bulk box, relieving one possible source of stresses for the inside vs. outside of the tubule.

15. Supplementary simulation data

WT, Comp. 1	Inner leaflet (mol %)				Outer leaflet (mol %)			
Replicate	SDPC	POPS	CHOL	PIP_2	SDPC	POPS	CHOL	PIP_2
1	57.4	19.6	21.2	1.8	58.4	17.0	16.0	8.6
2	57.4	19.3	21.1	2.2	58.4	17.2	16.0	8.4
3	59.3	17.7	20.4	2.6	57.2	18.2	16.5	8.1
4	59.0	19.3	20.2	1.6	57.4	17.2	16.6	8.8
5	58.5	19.1	20.4	2.0	57.7	17.3	16.5	8.5
6	59.4	18.5	20.9	1.2	57.1	17.7	16.2	9.0
7	61.4	14.8	20.4	3.4	55.9	20.0	16.5	7.6
8	59.2	17.1	20.4	3.4	57.3	18.6	16.5	7.7
9	56.8	19.1	20.9	3.2	58.7	17.3	16.1	7.8
10	56.9	19.9	21.5	1.6	58.7	16.8	15.8	8.7
Averages	58.5 ± 1.4	18.4 ± 1.5	20.7 ± 0.4	2.3 ± 0.8	57.7 ± 0.9	17.7 ± 1.0	16.3 ± 0.3	8.3 ± 0.5

F9A/F13A, Comp. 1	Inner leaflet (mol %)				Outer leaflet (mol %)			
Replicate	SDPC	POPS	CHOL	PIP_2	SDPC	POPS	CHOL	PIP_2
1	56.7	21.1	21.0	1.2	58.8	16.1	16.1	9.0
2	57.4	19.6	21.4	1.6	58.4	17.0	15.9	8.7
3	58.4	18.6	20.8	2.2	57.7	17.6	16.2	8.4
Averages	57.5 ± 0.9	19.8 ± 1.3	21.1 ± 0.3	1.7 ± 0.5	58.3 ± 0.6	16.9 ± 0.8	16.1 ± 0.2	8.7 ± 0.3

WT, Comp. 3	Inner leaflet (mol %)				Outer leaflet (mol %)			

Replicate	SDPC	POPS	CHOL	PIP_2	SDPC	POPS	CHOL	PIP_2
1	21.0	37.8	27.4	13.8	29.2	28.3	18.4	24.0
2	20.6	37.2	27.1	15.1	29.5	28.7	18.6	23.2
3	22.8	34.4	27.2	15.6	28.1	30.5	18.7	22.8
Averages	21.5 ± 1.2	36.5 ± 1.8	27.2 ± 0.2	14.8 ± 0.9	28.9 ± 0.7	29.2 ± 1.2	18.6 ± 0.2	23.3 ± 0.6

Table S7. Inner and outer leaflet compositions for all replicates of production simulations. Mean compositions and ± standard deviations of lipid mol % across replicates are in bottom rows.

	Thickness (Å)		
	Inner leaflet	Outer leaflet	Bilayer
WT CHMP1B + IST1 lipid composition 1	12.53 ± 0.08	14.89 ± 0.09	27.42 ± 0.15
F9A + F13A CHMP1B + IST1 lipid composition 1	12.63 ± 0.17	14.91 ± 0.06	27.54 ± 0.14
WT CHMP1B + IST1 lipid composition 17	13.29 ± 0.07	15.37 ± 0.11	28.66 ± 0.15
No-protein tubule lipid composition 1	12.74	14.82	27.56
Flat bilayer lipid composition 1	(Leaflets assumed to be identical)		30.50 ± 0.24

Table S8. Summary of leaflet and bilayer thicknesses from all simulations. Thicknesses are based on mean positions of the second glycerol bead for all phospholipids, and for tubules the location of the bilayer midplane. Tubule calculation procedures are described in SI 2.4, and tubule uncertainties are one standard deviation between calculated thicknesses of independent replicates. Full tubule structural data are in ED Figs. 2, 6-8. For the flat bilayer, a 250 x 250 Å patch was divided into a 250 x 250 grid, and for each frame a 2d cubic interpolation was applied over the grid to the positions of upper and lower leaflet second glycerol beads. A mean thickness was then calculated over the grid for each frame, and the mean over 200 frames from the last 400 ns of the simulation was calculated, with the uncertainty being the standard deviation across the 200 frames. Each of the lipid composition 1 (Table S6) tubules are around 10 % thinner than the composition 1 flat bilayer, with most of the thinning coming from the inner leaflet likely due to overall increased splaying of lipid tails in that leaflet. The lipid composition 17 (Table S6) tubules are slightly thicker, likely as a consequence of the composition being enriched in more rigid, saturated phospholipid tails relative to lipid composition 1 (Table S6).

16. References

1. Moss, F. R. *et al.* Ladderane phospholipids form a densely packed membrane with normal hydrazine and anomalously low proton/hydroxide permeability. *PNAS* **115**, 9098–9103 (2018).
2. Stottrup, B. L., Stevens, D. S. & Keller, S. L. Miscibility of ternary mixtures of phospholipids and cholesterol in monolayers, and application to bilayer systems. *Biophys J* **88**, 269–276 (2005).
3. Risselada, H. J. & Marrink, S. J. Curvature effects on lipid packing and dynamics in liposomes revealed by coarse grained molecular dynamics simulations. *Phys. Chem. Chem. Phys.* **11**, 2056–2067 (2009).
4. Nguyen, H. C. *et al.* Membrane constriction and thinning by sequential ESCRT-III polymerization. *Nat Struct Mol Biol* **27**, 392–399 (2020).
5. Heberle, F. A. *et al.* Direct label-free imaging of nanodomains in biomimetic and biological membranes by cryogenic electron microscopy. *Proc Natl Acad Sci U S A* **117**, 19943–19952 (2020).
