# Supplementary material for: Brominated lipid probes expose structural asymmetries in constricted membranes
Source: Nat Struct Mol Biol. 2023 Jan 9;30(2):167–75. doi: 10.1038/s41594-022-00898-1 (PMC9935397; doi:10.1038/s41594-022-00898-1)
Supplement: Supplementary file 2 — Reporting Summary [file 41594_2022_898_MOESM2_ESM.pdf]

nature portfolio

Corresponding author(s): Adam Frost and Michael Grabe

Last updated by author(s): Oct 31, 2022

Reporting Summary

Nature Portfolio wishes to improve the reproducibility of the work that we publish. This form provides structure for consistency and transparency in reporting. For further information on Nature Portfolio policies, see our Editorial Policies and the Editorial Policy Checklist.

Statistics

For all statistical analyses, confirm that the following items are present in the figure legend, table legend, main text, or Methods section.

n/a	Confirmed	
☐	☒	The exact sample size (*n*) for each experimental group/condition, given as a discrete number and unit of measurement
☐	☒	A statement on whether measurements were taken from distinct samples or whether the same sample was measured repeatedly
☒	☐	The statistical test(s) used AND whether they are one- or two-sided<br>*Only common tests should be described solely by name; describe more complex techniques in the Methods section.*
☒	☐	A description of all covariates tested
☐	☒	A description of any assumptions or corrections, such as tests of normality and adjustment for multiple comparisons
☐	☒	A full description of the statistical parameters including central tendency (e.g. means) or other basic estimates (e.g. regression coefficient) AND variation (e.g. standard deviation) or associated estimates of uncertainty (e.g. confidence intervals)
☒	☐	For null hypothesis testing, the test statistic (e.g. F, t, r) with confidence intervals, effect sizes, degrees of freedom and P value noted<br>*Give P values as exact values whenever suitable.*
☒	☐	For Bayesian analysis, information on the choice of priors and Markov chain Monte Carlo settings
☒	☐	For hierarchical and complex designs, identification of the appropriate level for tests and full reporting of outcomes
☒	☐	Estimates of effect sizes (e.g. Cohen's *d*, Pearson's *r*), indicating how they were calculated

Our web collection on statistics for biologists contains articles on many of the points above.

Software and code

Policy information about availability of computer code

Data collection	Electron microscopy data was collected using SerialEM 3.8. NMR data was collected with Bruker TopSpin 4. Langmuir trough isotherms were collected with KSV Nima Attension 2.3. Simulations were performed with Gromacs 2018.8 and Pymol 2.3.0.
Data analysis	Data was analyzed with MotionCor2, RELION 3.0.8, CTFFind4, ImageJ 1.53c, MestReNova 14.2.1, UCSF Chimera 1.15, Matlab R2019, Python 3.7.7, MDAnalysis 1.0.0, and SPHIRE-crYOLO 1.7.6. The software is referenced in the Methods section. Custom code is deposited with Zenodo (10.5281/zenodo.7232344).

For manuscripts utilizing custom algorithms or software that are central to the research but not yet described in published literature, software must be made available to editors and reviewers. We strongly encourage code deposition in a community repository (e.g. GitHub). See the Nature Portfolio guidelines for submitting code & software for further information.

Data

Policy information about availability of data

All manuscripts must include a data availability statement. This statement should provide the following information, where applicable:

- Accession codes, unique identifiers, or web links for publicly available datasets
- A description of any restrictions on data availability
- For clinical datasets or third party data, please ensure that the statement adheres to our policy

The cryo-EM maps have been deposited into the Electron Microscopy Data Bank (accession numbers EMD-27991, EMD-28694 – 28719, and EMD-28722). Motion-corrected micrographs have been deposited into the EMPIAR database (accession number EMPIAR-11277). PDB 6TZ5 was used to set up molecular dynamics simulations. Source data from figures is included with the manuscript.

Field-specific reporting

Please select the one below that is the best fit for your research. If you are not sure, read the appropriate sections before making your selection.

☒ Life sciences ☐ Behavioural & social sciences ☐ Ecological, evolutionary & environmental sciences

For a reference copy of the document with all sections, see nature.com/documents/nr-reporting-summary-flat.pdf

Life sciences study design

All studies must disclose on these points even when the disclosure is negative.

Sample size	Cryo-EM images were collected for each sample until there were sufficient sample views to perform 3D reconstructions at the desired resolutions. On the Krios, 1400-6000 micrographs were collected for each sample, resulting in 20,000 to 60,000 usable particles per sample and 2.8 to 4.3 Angstrom resolution reconstructions. On the Arctica, between 400 and 800 micrographs were collected for each sample, resulting in 1000 to 4000 usable particles per sample and 4 to 9.5 Angstrom resolution reconstructions. As the number of usable particles in each micrograph is highly variable due to differences in sample density, it was not possible to predetermine sample size. Simulations were run for 2.4 us after equilibration and were run at least 3 times independently.
Data exclusions	Particles from cryo-EM images were excluded from analysis if they were overlapping, damaged, nonuniform in filament diameter, or did not contain high resolution features. These exclusions were determined with 2D and 3D classification in RELION.
Replication	Biophysical analyses were repeated at least 3 times. Cryo-EM data were randomly divided into two halves that were independently refined. Simulations were repeated at least 3 times. All attempts at replicating the experimental and simulation data were successful.
Randomization	Data from each different sample is inherently randomized during cryo-EM data analysis. Particles are divided into random half sets and processed independently. Resolution estimates are based on comparison of independently processed half maps.
Blinding	Investigators were not blinded to sample identity, but were blinded with respect to the data randomization during cryo-EM data processing. All data sets from different samples were processed using the same parameters using a standardized data processing pipeline in RELION. Further blinding was not possible as CryoEM maps require a priori knowledge of the sample for interpretation. Investigators were not blinded to group allocation during other experiments and simulations as each sample was analyzed individually, not as part of a group.

Reporting for specific materials, systems and methods

We require information from authors about some types of materials, experimental systems and methods used in many studies. Here, indicate whether each material, system or method listed is relevant to your study. If you are not sure if a list item applies to your research, read the appropriate section before selecting a response.

Materials & experimental systems

n/a	Involved in the study
☒	☐ Antibodies
☒	☐ Eukaryotic cell lines
☒	☐ Palaeontology and archaeology
☒	☐ Animals and other organisms
☒	☐ Human research participants
☒	☐ Clinical data
☒	☐ Dual use research of concern

Methods

n/a	Involved in the study
☒	☐ ChIP-seq
☒	☐ Flow cytometry
☒	☐ MRI-based neuroimaging
